# Abnormal Gyrus Rectus Asymmetry in Alzheimer’s Disease: An MRI-Based Parcellation Method

**DOI:** 10.3390/brainsci15050452

**Published:** 2025-04-26

**Authors:** Ömür Karaca, Ahmet Arman Kibar, Burcu Aslantekin, Nermin Tepe

**Affiliations:** 1 Department of Anatomy, Faculty of Medicine, Balıkesir University, Balıkesir 10145, Turkey; armankibar69@gmail.com (A.A.K.); burcuaslantekin@gmail.com (B.A.); 2 Department of Neurology, Faculty of Medicine, Balıkesir University, Balıkesir 10145, Turkey; tepenermin@gmail.com

**Keywords:** brain asymmetry, Alzheimer’s disease, atlas-based method, gyrus rectus

## Abstract

Background: The gyrus rectus is a key brain region with neural connections to the entorhinal cortex and hippocampus, both of which are among the earliest areas affected in Alzheimer’s disease (AD). Investigating volumetric differences and asymmetry in this region may provide insights into disease progression. This study aimed to assess gyrus rectus volume and asymmetry in AD patients using an MRI-based parcellation method. Methods: This cross-sectional volumetric study included 25 cognitively healthy adults and 25 AD patients recruited from the Neurology Clinic of Balıkesir University Hospital. Brain MRI scans were obtained using a 1.5 Tesla MRI scanner. Volumetric measurements were computed using MRIStudio, an atlas-based image analysis program. Group differences in brain volume and asymmetry index were examined, and their correlations with Mini-Mental State Examination (MMSE) scores were evaluated. Results: AD patients exhibited significantly greater rightward volumetric asymmetry of the gyrus rectus volume than healthy controls (*p* < 0.05). Additionally, a positive correlation was observed between gyrus rectus volume and MMSE scores (*p* < 0.05). Conclusions: These results suggest that rightward volumetric asymmetry of the gyrus rectus may represent a promising biomarker for tracking the progression of Alzheimer’s disease. Detecting asymmetry in brain structures could improve understanding of AD pathology and aid clinical evaluation.

## 1. Introduction

Alzheimer’s disease (AD) is a progressive neurodegenerative condition that primarily affects the elderly and is characterized by specific clinical symptoms and distinct neuropathological changes. The key pathological features of AD include the buildup of extracellular amyloid-beta plaques and intracellular neurofibrillary tangles made up of hyperphosphorylated tau protein. These are widely recognized as the main histological indicators of the disease [1,2]. The neurodegenerative process in AD begins with the early impairment of synapses, followed by retrograde degeneration of axons, neuronal dysfunction, and, ultimately, neuronal cell death [1]. Neurodegenerative changes initially manifest in brain regions associated with memory processing, such as the entorhinal cortex and hippocampus, through the disruption of synaptic connectivity between neurons. It then spreads to other brain areas, such as the prefrontal cortex, which is responsible for decision making, cognitive functions, and social behavior [3,4].

Structural imaging studies have consistently revealed volumetric changes in the frontal lobe can be directly related to various neuropsychiatric symptoms of the AD including apathy, disinhibition, and agitation [5,6,7,8,9]. According to Cajanus et al., elevated disinhibition scores in AD patients were linked to reduced volumes in the ventromedial and lateral prefrontal regions, with the gyrus rectus being particularly affected [6]. Atrophy or volumetric asymmetries in the orbitofrontal cortex and prefrontal cortex have been observed with the progression of the disease and it has been demonstrated that the pathological progression of neurodegeneration does not affect the cerebral hemispheres equally [5,6,7,8,9]. Additionally, functional magnetic resonance imaging (MRI) studies in AD patients have suggested a relationship between orbitofrontal cortex atrophy and episodic recall and prospective memory difficulties [10]. These findings underscore the importance of region-specific analyses in understanding the disease’s progression and its clinical correlates.

Within the frontal lobe, the gyrus rectus (a subregion often considered part of the orbitofrontal cortex) has received increasing attention for its potential role in AD pathology. Anatomically, the orbitofrontal cortex is subdivided into three distinct components: the gyrus rectus, the medial orbitofrontal gyrus, and the lateral orbitofrontal gyrus [11,12]. The gyrus rectus is situated medial to the olfactory sulcus on the inferomedial surface of the frontal lobe and has been accepted as the frontal extension of the anterior cingulate gyrus [13,14]. However, functional imaging studies in individuals with schizophrenia have revealed a distinct functional differentiation between the orbitofrontal cortex and the gyrus rectus during various cognitive tasks. These findings suggest that the gyrus rectus may be involved in a specialized neural network responsible for specific aspects of emotional processing in humans [11,13]. For instance, a study of patients undergoing gyrus rectus resection reported temporary postoperative deficits in short-term recall and verbal fluency, with subsequent recovery, pointing to its functional significance [14]. Although the morphometric changes in the orbitofrontal cortex have been extensively investigated in AD, the gyrus rectus has received comparatively less interest, despite its potential relevance to the disease’s cognitive.

The aim of this study was to investigate the volume differences and degree of asymmetry of the gyrus rectus in patients with AD using a reliable automatic volume calculation method. Examining asymmetrical variations in brain structures offers valuable insights into the etiology, clinical characteristics, and progression of neurodegenerative diseases. Indeed, asymmetry-based assessments may prove to be more informative than absolute bilateral measurements in the diagnostic evaluation of such conditions.

## 2. Materials and Methods

### 2.1. Subjects

Twenty-five mentally healthy adults (age: 62.16 ± 8 years old M ± SD) and twenty-five AD patients (age: 69.20 ± 7 years old M ± SD) were recruited to the study. All participants were selected from among patients who applied to the neurology clinic of Balıkesir University Hospital. Right-hand dominant individuals were included in the study. A neurologist examined all participants and diagnosed AD according to diagnostic criteria determined by the National Institute of Neurological and Communicative Disorders and Stroke and Related Disorders Association for Alzheimer’s disease [15]. All participants underwent the Mini-Mental State Examination Score (MMSE) test to assess memory loss. Participants with concurrent neurological and psychiatric diseases, vitamin B12 deficiency, diabetes, or hematological/oncological disorders were excluded from the study. The study was conducted in accordance with the Declaration of Helsinki and received approval from the Balıkesir University Health Sciences Non-Interventional Ethics Committee (document number: 2018/87) on 25 April 2018. Informed consent was obtained from each study subject. If the patient did not have the capacity to give consent, written consent is obtained from his/her relatives.

### 2.2. MRI Acquisition

All participants, including patients and controls, underwent the same imaging protocol using a 1.5 Tesla MRI system (Philips Healthcare in Best, Ingenia, 2013, Wormerveer, The Netherlands) to acquire whole-brain T1-weighted images. Coronal T1-weighted images were obtained with turbo spin echo sequences, with the following parameters: repetition time (TR) of 7.0 ms, echo time (TE) of 3.4 ms, a field of view (FOV) of 250 × 250 mm, and a matrix size of 228 × 228. The voxel dimensions were set to 1 × 1 × 1 mm, with a flip angle 90°. The slice thickness was 1.1 mm, with no gap between slices.

### 2.3. Volumetric Analysis on MRI

#### 2.3.1. Brain Parcellation with MRIStudio (Atlas-Based Method)

**Figure 1:**
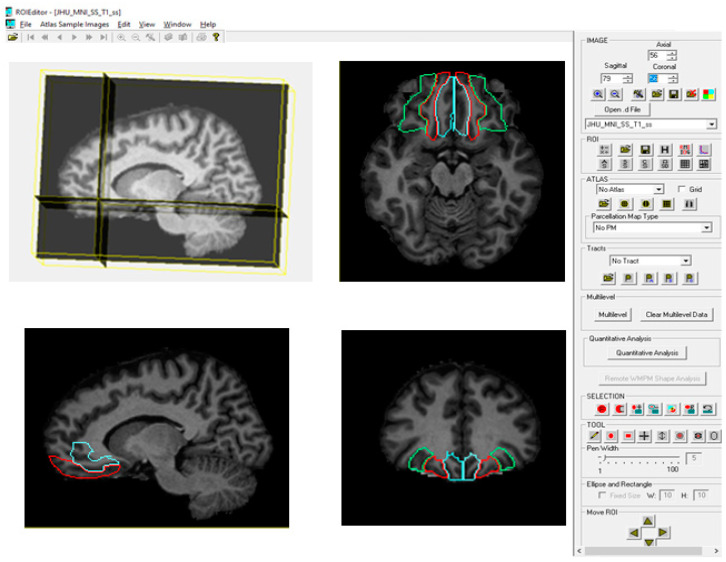
Identification of the gyrus rectus (blue), medial orbitofrontal gyrus (red), and lateral orbitofrontal gyrus (green) on normalized MRI images using ROI Editor.

We assessed the volume and asymmetry index of the following brain structures: the brain hemispheres, the gyrus rectus (Brodmann Area 14, BA14), the lateral orbitofrontal gyrus (BA11), and the medial orbitofrontal gyrus (BA12) (Figure 1).

In this study, brain parcellation was carried out using MRIStudio 1.8, a software application developed by Johns Hopkins University and the Kennedy Krieger Institute. This suite consists of three core programs: DtiStudio, DiffeoMap, and RoiEditor, which are used in sequence for image acquisition, preprocessing, normalization, and automated anatomical segmentation. MRIStudio employs an atlas-based approach to ensure standardized brain parcellation across subjects. The software is publicly available at http://www.MRIStudio.org (accessed on 3 May 2019). 

#### 2.3.2. Image Acquisition and Preprocessing

In this study, DtiStudio was employed to retrieve and process DICOM (Digital Imaging and Communications in Medicine) images obtained from each participant. The raw MRI data were evaluated for artifacts, motion distortions, and intensity inhomogeneities to ensure robust preprocessing. Necessary corrections, including eddy current compensation and intensity normalization, were implemented prior to subsequent processing.

#### 2.3.3. Image Registration and Normalization (DiffeoMap Processing)

To enable inter-subject comparisons, MRI data from each participant underwent registration and normalization using the DiffeoMap software. This process was crucial for aligning individual brain structures to a shared anatomical space. The procedure involved several steps. Non-brain tissues, including the skull, dura mater, and cerebrospinal fluid artifacts, were automatically removed to enhance segmentation accuracy through skull-stripping. An initial registration was then applied to align each participant’s MRI scan with a standardized brain template via affine transformation. Subsequently, a high-resolution nonlinear transformation was conducted using the Large Deformation Diffeomorphic Metric Mapping (LDDMM) algorithm to achieve precise spatial normalization, ensuring mapping of anatomical features while preserving structural integrity. Finally, the resulting images were aligned with a predefined skull-stripped template to ensure consistency across participants.

#### 2.3.4. Automated Brain Parcellation (RoiEditor Processing)

Following the normalization of MR images, the RoiEditor software was utilized to conduct image analysis based on a single atlas, resulting in the segmentation of the brain into 189 anatomical structures. This atlas-based approach enabled automated and reproducible parcellation of cortical and subcortical regions. The atlases used for volumetric analysis in MRIStudio software allow for specific frontal lobe segmentation, including a detailed segmentation of the rectus gyrus (Figure 1). Commonly referenced atlases, such as JHU-MNI, JHU-Talairach, and ICBM-MNI, are documented in the RoiEditor user manual (MRIStudio User Manual), and the method employed in this study adheres to a well-established neuroanatomical framework, ensuring that all segmented brain regions align with recognized anatomical landmarks. Regions of interest (ROIs) were extracted and assigned according to predefined neuroanatomical criteria, facilitating robust volumetric analysis. Subsequently, the volume of each region was automatically calculated by multiplying the number of voxels assigned to that region by the voxel volume, allowing for quantitative evaluation of brain structures. The MRIStudio-based parcellation approach applied in this study has been thoroughly validated in prior research, confirming its accuracy, reproducibility, and suitability for neuroanatomical investigations [16,17].

#### 2.3.5. Asymmetry Index (AI)

The asymmetry index (AI) was calculated using the formula AI = [(L − R)/0.5 (L + R)] × 100%, where L and R represent the volumes of the left and right brain structures, respectively [18]. According to this formula, a positive AI indicates that the left hemisphere is larger than the right, reflecting left-lateralized asymmetry, while a negative AI suggests that the right hemisphere exceeds the left, indicating right-lateralized asymmetry [18].

### 2.4. Statistical Analysis

Statistical analyses were performed using the Statistical Package for the Social Sciences (SPSS) software, version 16.0 for Windows (SPSS Inc., Chicago, IL, USA) [19]. All volumetric values were reported as mean ± standard deviation (SD). Before proceeding with the analyses, the data distribution was examined using the Shapiro–Wilk test to verify the suitability for parametric testing. Brain volumes and asymmetry index values between AD patients and healthy controls were compared using paired *t*-tests. The effect size for these comparisons was determined by calculating Cohen’s d, which measures the magnitude of group differences. Pearson’s correlation coefficient (r) was also applied to investigate the association between volumetric measures and MMSE scores, assessing the link between brain volume alterations and cognitive impairment. Correlation strength was categorized as weak (0.1–0.3), moderate (0.3–0.5), or strong (≥0.5). Additionally, the effect of age on brain regions was assessed using ANCOVA. The results of the analysis of covariance (ANCOVA) conducted to determine the impact of age on volumetric differences indicated that age did not have a statistically significant effect on the relevant brain volumes (*p* > 0.05). For all statistical analyses, a significance level of *p* < 0.05 was considered indicator of statistical significance.

## 3. Results

The mean values of demographic characteristics, including age, sex distribution, and MMSE scores, are presented in Table 1.

**Figure 2:**
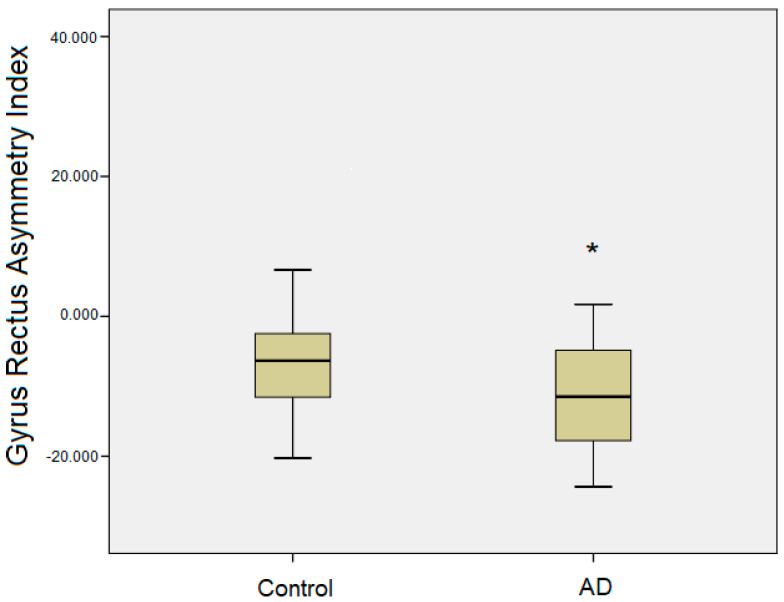
Boxplots illustrating the degree of gyrus rectus asymmetry in healthy controls and AD patients. The x-axis displays the asymmetry index (AI), with negative values indicating greater volume in the right hemisphere compared to the left. * Indicates statistical significance at *p* < 0.05.

The volumetric measurements of the examined brain structures, along with the calculated asymmetry index values, are presented in Table 2. In the AD group, there was no evidence of left-sided atrophy in the left gyrus rectus volume. Although a relative increase in the right gyrus rectus volume was observed compared to healthy controls, this difference did not reach statistical significance. The only statistically significant finding was observed in the asymmetry index values, which indicated a greater rightward asymmetry in the AD group (*p* < 0.05) (Table 2). Post hoc analyses confirmed this pattern, showing significantly increased rightward volumetric asymmetry of the gyrus rectus in AD patients compared to healthy controls, suggesting an asymmetric alteration in this region associated with AD pathology (Figure 2). In contrast, no statistically significant volumetric differences or asymmetry changes were detected in other regions of the orbitofrontal cortex (Table 2).

Furthermore, correlation analyses demonstrated a significant positive association between the volumetric measurements of the bilateral gyrus rectus and MMSE scores (*p* < 0.05) (Table 3). Additionally, a significant positive correlation was identified between the gyrus rectus volumes and the total orbitofrontal cortex volumes (*p* < 0.05) (Table 4), indicating a potential interdependence between these structures in the context of neurodegeneration.

## 4. Discussion

Structural and functional asymmetries in the human brain are increasingly recognized as significant prognostic indicators for various neurological disorders, including AD. In the present study, we investigated the volumetric asymmetry of the gyrus rectus and found a statistically significant rightward asymmetry in AD patients compared to healthy controls.

The gyrus rectus, located on the inferior surface of the frontal lobe, medial to the olfactory sulcus, is anatomically positioned at the interface of multiple neural networks. It has connections with the limbic system and olfactory regions through the anterior cingulate gyrus [20]. The anterior cingulate cortex is a component of the frontal–subcortical neural circuits, primarily receiving afferent connections from the hippocampus. The hippocampus, in turn, communicates with higher-order associative cortical areas through the entorhinal, perirhinal, and parahippocampal regions [11,21]. The parahippocampal gyrus has an important function in memory formation, especially in the recall of visual scenes. Zhu et al. (2019) demonstrated that disrupted connectivity between the parahippocampal gyrus and orbitofrontal cortex may serve as an early biomarker of AD progression, underscoring the relevance of these networks in disease pathology [22].

In a study where visual memory functions were tested to reveal the function of the gyrus rectus, a greater increase in activation was observed in the right medial orbitofrontal cortex, which includes the gyrus rectus, compared to the left side. It has been stated that right-sided activity is associated with the processing of icon-like images, which are more difficult to retain in memory for longer periods of time, whereas the left side is associated with the retention of more durable categorical (or verbally labeled) representations [23,24,25]. Additionally, studies comparing patients with right and left hemispheric lesions have shown that damage to the right gyrus rectus results in more severe memory impairments, highlighting its essential role in encoding new information and supporting short-term memory processes [11,24,25]. These hemispheric differences emphasize the gyrus rectus’s specialized contributions to memory and its potential vulnerability in neurodegenerative disease like AD.

Given its anatomical and functional significance, the gyrus rectus emerges as a region of interest in AD, particularly in light of the asymmetric brain changes documented in this disorder. Volumetric asymmetries in AD have been well established in regions such as the medial temporal lobe, hippocampus, amygdala, and prefrontal cortex [26,27,28,29,30]. For example, Kim et al. (2012) reported rightward asymmetry in frontal lobe cortical thickness among AD patients, suggesting a regional susceptibility to AD pathology [27]. Similarly, our previous research demonstrated abnormal rightward asymmetry in the dorsolateral prefrontal cortex in AD patients compared to controls [28]. Our results of increased rightward asymmetry in the gyrus rectus are compatible with previous studies and support a broader pattern of right-hemisphere vulnerability that includes both temporal and frontal regions in AD.

In mild cognitive impairment (MCI) patients with two years of follow-up, it was found that cortical thinning in the right medial orbitofrontal cortex increased in proportion to increased amyloid plaque deposition in the right superior temporal gyrus and visual memory weakened over time in these patients [26]. While these findings indicate cortical thinning in the right gyrus rectus, they support the view that both volumetric and functional changes in the gyrus rectus and its connected regions are strongly associated with visual memory deficits. Notably, to our knowledge, no prior studies have directly examined gyrus rectus volumetric asymmetry using MRI in AD patients, positioning our research as a novel contribution to this field.

Several studies reported that the pathological features of AD, including Aβ plaque distribution, follow an asymmetrical pattern that varies with the disease stage, and that this asymmetry is more pronounced in the MCI stage than in advanced AD stages [31,32,33]. This suggests that the distribution of Aβ plaques gradually becomes asymmetrical during AD progression. In addition, it has been shown that Aβ deposition is significantly more significant in the right superior parietal gyrus, inferior parietal gyrus, and ventromedial prefrontal cortex (including gyrus rectus) than in the corresponding regions of the left hemisphere during the AD stage with similar asymmetries already evident in the ventromedial prefrontal cortex during the MCI stage [32]. Supporting this, previous research has demonstrated that the distribution of Aβ fibrils tends to follow the functional and anatomical architecture of the default mode network (DMN), with asymmetrical accumulation patterns, particularly in the right hemisphere, becoming more apparent during the MCI and early AD stages [33]. Brain regions connected by DMN are among the earliest affected in AD, with the progression of pathological changes closely following the pathways of the DMN. The posterior DMN mainly includes the posterior cingulate cortex, precuneus, bilateral angular gyrus, and bilateral middle temporal gyrus. In contrast, the anterior DMN consists of the superior frontal gyrus, the superior medial gyrus, the anterior cingulate cortex, and the ventromedial prefrontal cortex (including the gyrus rectus) [33]. These patterns may reflect asymmetric Aβ accumulation that aligns with the structural and functional organization of the DMN and progresses in parallel with neurodegeneration. The mean MMSE score in our AD group was 18, reflecting the early stage of Alzheimer’s disease in these patients. In this context, the more pronounced rightward volumetric asymmetry of the gyrus rectus identified in our study may represent the asymmetric spread of neurodegeneration along the DMN and the preferential lateralization of Aβ pathology.

The observed rightward asymmetry in the gyrus rectus may reflect compensatory mechanisms mobilized in response to neurodegenerative changes [34,35,36]. Liang et al. (2011) proposed that MCI patients recruit additional neural resources in the right prefrontal cortex to compensate for cognitive deficits, with progressive structural and functional impairments in limbic structures potentially driving neuroplasticity in prefrontal regions [34]. Therefore, this result can be interpreted as an attempt to recruit the right prefrontal cortex in semantic and episodic tasks. This compensatory recruitment may disrupt the brain’s normal lateralization patterns, leading to abnormal right lateralization as a protective strategy against memory loss. In healthy aging, lateralization supports efficient cognitive processing, with the left hemisphere typically dominating language and semantic functions, and the right hemisphere excelling in visuospatial and emotional processing [35]. In AD, however, this balance appears to shift, with increased right hemisphere engagement potentially reflecting an attempt to preserve function in the case of widespread neurodegeneration. Functional imaging studies have reported similar compensatory patterns, such as increased right prefrontal activation during memory tasks in MCI and early AD patients [36].

An alternative, yet complementary, perspective assumes that the right prefrontal cortex, including the gyrus rectus, contributes to the suppression of unwanted memories [37,38,39,40]. Researchers have indicated that intentional forgetting occurs through inhibition-related activity in the right prefrontal cortex [38,39]. In particular, the right ventrolateral prefrontal cortex plays an inhibitory role in the downregulation of negative emotions while the left ventrolateral prefrontal cortex mainly serves as a semantic processor that generates and selects appropriate evaluations [39]. In a related study, Lu et al. (2022) found a significant positive correlation between the gray matter volume of the right superior frontal gyrus and thought suppression in healthy adults [40]. Considering its function and connections, volumetric rightward asymmetry of the gyrus rectus may also underlie difficulties in the suppression of retrieval, as may be observed in AD.

Despite the important findings of this study, several limitations should be noted. First, the relatively small sample size limited our statistical power and ability to control for experiment-wise error rates, increasing the risk of type I errors. Second, we were unable to incorporate functional MRI assessments to evaluate brain connectivity and volume–function relationships, which would have provided further insights into the role of the gyrus rectus in AD pathogenesis.

## 5. Conclusions

In this study, the observed rightward volumetric asymmetry of the gyrus rectus in individuals with AD may represent either a compensatory response to underlying structural degeneration or a mechanism associated with suppressing unwanted memories. Advanced neuroimaging techniques provide valuable opportunities to detect structural and functional alterations in the gyrus rectus, offering insights into the neuropathological processes of AD. Nevertheless, further comprehensive and longitudinal studies are needed to clarify whether this abnormal rightward asymmetry could be a reliable biomarker for monitoring disease progression and predicting clinical outcomes in patients with AD. Identifying such markers would contribute significantly to improving diagnostic accuracy and guiding therapeutic interventions in the clinical management of AD.

## Figures and Tables

**Table 1:** Comparison of demographic data and MMSE scores between AD patients and healthy controls.

	AD Patients	Controls	F	*p*
No. of subjects	25	25		-
Age (yr)	69.20 ± 7.7 (56–80)	62.16 ± 8.2 (52–80)	0.059	0.003
Women/men	(20/5)	(18/7)		-
MMSE score	18.08 ± 5.7	29.48 ± 0.5	23.701	0.001

AD: Alzheimer’s disease, MMSE: Mini-Mental State Examination.

**Table 2:** Volumetric analysis of brain structures and asymmetry index.

Brain Regions	Volume (cm^3^) (Mean ± SD)					
	Controls			AD Patients		
	Left	Right	AI (L-R)	Left	Right	AI (L-R)
Hemisphere	545.1 ± 66	551.8 ± 69	−1.15 ± 2.5	517.3 ± 50	525.5 ± 50	−1.59 ± 3.0
Gyrus Rectus	6.07 ± 1.6	6.47 ± 1.8	−4.26 ± 11	6.00 ± 1.6	6.78 ± 1.8	−11.82 ± 7.7 *
Lateral OFC	6.12 ± 1.8	6.87 ± 2.1	−7.66 ± 21	6.35 ± 1.5	7.23 ± 1.6	−12.71 ± 12
Medial OFC	5.13 ± 1.6	4.64 ± 1.4	12.8 ± 22	5.16 ± 1.3	4.68 ± 1.1	7.7 ± 13

* Indicates a significant increase (*p* < 0.05) in rightward asymmetry of the gyrus rectus in AD patients compared to healthy controls, as determined by a *t*-test. AI: asymmetry index; OFC: orbitofrontal cortex.

**Table 3:** Pearson’s correlation coefficients between volumetric parameters and MMSE scores in AD patients.

Correlation to MMSE					
Left	r	*p*	Right	r	*p*
Hemisphere	0.621	0.001 *	Hemisphere	0.530	0.006 *
Gyrus Rectus	0.398	0.049 *	Gyrus Rectus	0.410	0.042 *
Lateral OFC	0.302	0.143	Lateral OFC	0.274	0.185
Medial OFC	0.257	0.214	Medial OFC	0.329	0.108

* Indicates significant positive correlations between volumetric parameters and MMSE scores. OFC: orbitofrontal cortex.

**Table 4:** Correlation analysis of gyrus rectus and orbitofrontal cortex volumes in AD patients.

Brain Regions	Left Lateral OFC		Right Lateral OFC		Left Medial OFC		Right Medial OFC	
	r	*p*	r	*p*	r	*p*	r	*p*
Left Gyrus Rectus	0.775	0.000 *	0.933	0.000 *	0.894	0.000 *	0.920	0.000 *
Right Gyrus Rectus	0.823	0.000 *	0.919	0.000 *	0.889	0.000 *	0.941	0.000 *

* Indicates significant positive associations between the volumes of the gyrus rectus and orbitofrontal cortex regions. OFC: orbitofrontal cortex.

## Data Availability

The original contributions presented in this study are included in the article. Further inquiries can be directed to the corresponding author.
